# Considerations for Applied Behavior Analysts Embracing Intersectional Qualitative Research Within the Context of Intervention Research

**DOI:** 10.1007/s40617-025-01104-6

**Published:** 2025-12-05

**Authors:** Sophia R. D’Agostino, Sarah E. Pinkelman

**Affiliations:** 1 Department of Special Education and Rehabilitation Counseling, Utah State University, 2865 Old Main Hill, Logan, UT USA; 2 Department of Psychology, Western Michigan University, Kalamazoo, MI USA

**Keywords:** Qualitative inquiry, Intersectionality, Behavior analysis

## Abstract

This is a pivotal time for researchers in applied behavior analysis (ABA) to embrace intersectional qualitative methods as ABA has faced criticism for its use of aversive procedures, emphasis on compliance, and focus on normalization, which can be detrimental to autistic individuals and other marginalized groups. It is imperative ABA shifts and adjusts to remain relevant and meaningful to the people we aim to support while encouraging social justice for all. Intersectional qualitative inquiry allows us to uncover how systems of privilege and oppression affect those who experience them: that is, how an individual’s interactions with these systems produce their learning history. Better understanding an individual’s learning history by examining intersectionality through qualitative inquiry allows us to integrate social justice and equity more deeply into ABA work. Applied behavior analysts must critically evaluate their practices and adopt approaches that respect and acknowledge the lived experiences of individuals, especially those from marginalized communities. This conceptual manuscript outlines considerations for embracing intersectional qualitative inquiry, including ways to begin thinking intersectionally that align with behavior analysis and of how intersectionality applies to each stage of the research process.

It is a critical time for the field of behavior analysis to consider intersectional qualitative methods within the context of intervention research. Applied behavior analysis (ABA) has faced scrutiny regarding the use of aversive procedures, emphasis on compliance, and a focus on normalization (Sandoval-Norton et al., 2019; Shyman, 2016) that can be harmful to autistic individuals (Anderson, 2023; Bottema-Beutel et al., 2021) and other marginalized groups. Over the past 50 years, several foundational and influential papers have focused on the social importance of behavior-analytic work to society. When considering the “society” that is impacted by ABA, we can consider the varying cultures in society. Prior behavior-analytic literature has conceptualized culture as a dynamic network of individual behavior that forms collective patterns where the behavior of one individual influences and is influenced by the behavior of others (interlocking behavioral contingencies; IBCs; Glenn, 1988; 2004). As a result of these IBCs, collective outcomes are produced (aggregate products; Glenn & Malott, 2004) that would otherwise not be achieved by uncoordinated, individual behavior. We could consider behavior analysts serving different or multiple “societies” with varying cultural practices depending on the unit of interest (e.g., global society, U.S. society, local communities, schools). Given the contextual nature of societies, culture, and individuals nested in these units and aspirations to better our world with behavior analysis (Malott, 1992; Skinner, 1948; 1987), prior literature has called attention to the social validity of ABA practices (Fawcett, 1991a; Schwartz & Baer, 1991; Wolf, 1978), the applied nature of ABA research (Baer et al., 1968; Fawcett 1991b), our humility (Kirby et al., 2022; Neuringer, 1991), and the personal liberties and quality of life of the individuals we serve (Bannerman et al., 1990; Miller et al., 2019; Schwartz & Kelly, 2021). Applied behavior analysis has also been somewhat influenced by constructional approaches and calls for prioritizing compassion in behavior change efforts (Goldiamond, 1974; Scallan & Rosales-Ruiz, 2023; Taylor et al., 2019). Although these efforts have led to improved practice and research, we cannot ignore the persistent urging from societies and voices within the ABA community to make further improvements. We believe that embracing intersectional qualitative research can support ABA in improving its cultural responsiveness, ethical rigor, and efficacy by better understanding the diverse and intersecting identities of the individuals and communities served by applied behavior analysts. This conceptual manuscript proposes an intersectional approach to qualitative methods in ABA intervention research.

While referring to recommendations from prior scholars (e.g., Esposito & Evans-Winters, 2021; Fawcett, 1991a,b; Leadbitter et al., 2021), we unpack ways in which an intersectional approach to qualitative research can support ABA researchers in systematically examining intersectionality and incorporating inclusive and equitable practices throughout the research process. For applied behavior analysts, to be truly “applied” means to be in pursuit of social justice (Pritchett et al., 2022). As one way to incorporate this value into our scientific endeavors, we propose embedding intersectionality into qualitative research endeavors as well as quantitative intervention research methods (and intervention research more broadly).

## What is Intersectional Qualitative Research?

Intersectionality is a term originally proposed by Crenshaw (1989) to describe intersecting systems of oppression that impinge upon the rights of an individual or group rather than treating these systems/categories as mutually exclusive. Examples of these intersecting categories (and the systems that produce oppression of people from these categories) include but are not limited to race, ethnicity, sexual orientation, gender, gender identity and expression, ability, neurodivergence, religion, education, citizenship status, and socioeconomic status. In qualitative research, applying an intersectional lens means exploring these overlapping identities and systems of oppression or privilege in context, allowing researchers to capture the complexity of participants’ experiences beyond single-dimensional analyses (Collins, 2015; Hancock, 2007). This approach emphasizes understanding the multifaceted and situated nature of social realities, which traditional methods, such as single case designs, may overlook. Applying intersectionality in qualitative research means taking seriously the identities people hold and how these complex identities intersect to shape their experiences of power, oppression, privilege, and marginalization (Crenshaw, 1991). Doing intersectional qualitative inquiry means more than just mentioning these identities; it involves integrating intersectionality into the research design, analysis, and interpretation in meaningful and systematic ways (Christensen & Jensen, 2012).

Intersectionality is applied in qualitative research across various fields, including special education (Garcia & Ortiz, 2013), healthcare (Abrams et al., 2020; Holman et al., 2021), developmental science (Santos & Toomey, 2018), and the broader social sciences (Esposito & Evans-Winters, 2021). More recently, intersectionality is also being incorporated into quantitative research (Bauer et al., 2021). Critical quantitative (QuantCrit) brings an intersectional lens to quantitative research, when in the past intersectionality was incorporated primarily in qualitative approaches (Bauer et al., 2021; Vargas & Peet, 2024). See Cruz et al. (2021) for an example of how dis/ability critical race theory (DisCrit) and QuantCrit were used to analyze a quantitative data set. In this conceptual manuscript, we focus on intersectional qualitative inquiry in the field of ABA.

### Why Should ABA Researchers Consider Intersectional Qualitative Inquiry?

Description, prediction, and control are goals of behavior analytic work, and each can be achieved with greater precision through an intersectional lens. As behavior analysts, we understand the importance of learning history in achieving these goals. This learning history is established through interactions between an individual’s behavior and their environment. Overlaying an intersectional lens to this conceptualization reveals how individuals will experience different behavior–environment interactions by nature of who they are. These behavior–environment interactions produce an individual’s learning history, and the resulting learning history for one individual will look different than another. Intersectionality teaches us that individuals experience diverse systems of power and oppression, and that the dynamic interplay between behavior and these environmental systems directly shapes and constitutes their learning history. Once we acknowledge the interactions between behavior and environment comprise the learning history, the challenge then becomes how to better understand an individual’s learning history, given that we cannot directly observe the behavior–environment interactions throughout an individual’s life that make up their learning history.

Intersectional qualitative inquiry allows us to uncover how systems of privilege and oppression affect those who experience them and how an individual’s interactions with these systems produce their learning history. Although intersectional qualitative research itself does not allow for the description, prediction, and control of behavior, it does provide complimentary information to assist us in better understanding a person’s learning history. When we are better able to describe a person’s learning history, we are better able to operationally define the behavior, predict the conditions under which behavior is more and less likely to occur, and manipulate environmental variables to affect dimensions of a particular behavior.

Better understanding an individual’s learning history by examining intersectionality through qualitative inquiry allows us to integrate social justice and equity more deeply into ABA work. This echoes prior calls for the need for diversity, equity, and inclusion and cultural humility in ABA practice (e.g., Fong et al., 2016; Wright, 2019) and research (see Cox et al., 2022; Pritchett et al., 2022) to address disparities and improve outcomes for those who receive our services. In the United States, disparities exist across most (if not all) systems in our society. People from minoritized groups (e.g., race, ethnicity, gender, sexual orientation) experience oppressive systems in areas such as wealth, income, education, health, housing, and criminal justice. Through intersectional qualitative inquiry, we are better able to uncover how these systems impact participants, as their lived experience can impact their experience with behavioral interventions. Relatedly, research ethics and diversity, equity, and inclusion in the research process (see Cox et al., 2022; Pritchett et al., 2022) is another reason that applied behavior analysts could benefit from intersectional qualitative inquiry.

All applied behavior analysts who hold certification or have completed an application for certification must act in accordance with the Ethics Code for Behavior Analysts (BACB, 2020), including their professional research activity. Core principles identified in the Code include, maximizing the benefits to others and treating others with compassion, dignity, and respect. Standard 1.08: Nondiscrimination stresses the importance of treating others in an equitable and inclusive manner. Cultural Responsiveness and Diversity (Standard 1.07) calls for us to evaluate our biases and our ability to address the needs of individuals with diverse needs and backgrounds. Relatedly, Awareness of Personal Biases and Challenges (Standard 1.10) requires that we maintain awareness of personal biases that could interfere with our work and take necessary steps to resolve interference to ensure that our work is not compromised, documenting actions taken. Involving clients and stakeholders is also identified in the Code (Standard 2.09). These core principles and standards align with the need to embrace diversity and collaborate when taking an intersectional approach to research.

Additionally, ABA lacks diversity in comparison to the extremely diverse societies it serves. This can result in cultural mismatch where the researcher’s cultural background differs from the culture of participants. To illustrate, most members of the Association for Behavior Analysis International (ABAI) in 2023 identified as white (71%), heterosexual (72%), and cisgender female (68%; ABAI, n.d.). Diversity among professionals in the field of ABA and the pursuit of equitable service delivery remain ongoing and much-needed areas of discussion. Although the field appears to value cultural competency (Fong et al., 2016), there remains a lack of culturally relevant behavior-analytic knowledge and professional training materials (Fong & Tanaka, 2013).

Our pursuit of improving human life does not occur in a vacuum; our work occurs in the context of the social systems and culture in which human life occurs. As applied behavior analysts we acknowledge that the environment in which behavior occurs reflects a rich, critical context for the analysis of behavior. As such, it is imperative that ABA shifts and adjusts to remain relevant and meaningful to the people we aim to support. One recent shift in ABA is the push for greater emphasis on the lived experiences of autistic individuals and individuals from other marginalized groups (e.g., DePape & Lindsay, 2016). As applied intervention researchers, we posit that we should follow the advice of Baer et al. (1968) that our research “is not determined by the research procedures used but by the interest which society shows in the problems being studied” (p. 92). Although single case design (SCD) research methodology is predominantly the method behavior analysts are trained to employ (e.g., section D of the BACB Test Content Outline, 6th ed.; BACB, 2024) and the methodology most commonly used in behavior analytic journals, SCD may not be the method that answers the questions society is interested in. To understand what society is interested in, applied behavior analysts are called to prioritize input from clients and invested parties, aligning with the principles of community-based participatory research (CBPR), which emphasizes collaboration, co-learning, and shared decision-making with community members throughout the research process (Fawcett 1991b; Watson-Thompson & Thompson, 2023). This is not a new practice; the call to focus on social validity has existed for decades (Kazdin, 1978; Schwartz & Baer, 1991; Wolf, 1978). However, more recent events impacting society have highlighted the importance of attending to society’s voices (e.g., COVID-19 Pandemic; Nicolson et al., 2020; Ethical concerns with ABA from autistic adults; Baiden et al., 2024; Police brutality in black communities; Alang et al., 2022). Despite these events, a focus on qualitative research appears to be lacking and the need to go beyond assessing social validity is warranted. Using methods that provide qualitative data leads to different kinds of knowledge than that from quantitative data. This knowledge production can allow intervention researchers to deeply explore intersectional lived experiences and perceptions, which can allow for valuable insight beyond the effectiveness of the intervention as measured quantitatively.

Qualitative research is historically interdisciplinary and informed by many genres and theoretical perspectives (Esposito & Evans-Winters, 2021). Therefore, the defining features of qualitative research can vary considerably. In comparison to quantitative research, we can define qualitative research as:

a form of social inquiry that tends to adopt a flexible and data-driven research design, to use relatively unstructured data, to emphasize the essential role of subjectivity in the research process, to study a small number of naturally occurring cases in detail, and to use verbal rather than statistical forms of analysis (Hammersley, 2012, p.12).

Some view quantitative and qualitative research as differing methods that can be used to answer research questions, while others see quantitative and qualitative approaches encompassing divergent worldviews, ways of knowing, or paradigms (Hammersley, 2012). Although we acknowledge behaviorism is not a fixed or singular position (i.e., undergirded by more than one paradigm), the positivist paradigm is persistent within the field due to core philosophical assumptions that prioritize quantitative inquiry (Morris, 2003). A shift in ABA to include more ways of knowing may depend on broadening our world view and methodological philosophies before simply adding qualitative research to ABA practice. Therefore, we outline some basic assumptions proposed by Esposito and Evans-Winters (2021) that serve as foundational premises for embracing an intersectional qualitative approach that we hope will be helpful to applied behavior analysts.

## Considerations for Embracing an Intersectional Qualitative Research Approach

### Claims We Must Accept to Think Intersectionally

Esposito and Evans-Winters (2021) propose claims that we must accept to think intersectionally. We want to highlight these claims as ways to promote intersectionality within the context of ABA intervention research. These claims are in surprising alignment with behavior analysis, and we point out some of these similarities below.

### Our Lived Experiences Shape Us

Esposito and Evans-Winters (2021) attest that we first must accept that our lived experiences shape us and therefore our approach to the research process. This assumption is in alignment with radical behaviorism (Schneider & Morris, 1987; Skinner, 1945) in that the study of behavior is a natural science that examines the impact of the environment on behavior. Similar to how a child’s past and current environments govern their behavior at school, a researcher’s lived experiences affect their scholarly behavior. A researcher’s scholarly behavior is not simply a product of their academic training and lived experiences. Their scholarly behavior is also shaped by their behavior–environment interactions outside of the formal classroom. By reflecting on this truth, we can acknowledge how our behavior as a scientist should undergo consistent reflection and evaluation to ensure that we are behaving in a manner that is in accordance with our ethics and values. Intersectionality, as a methodology, requires the researcher to consistently engage in reflection with ourselves and others concerning our power in the research process. We must reflect on how our experiences have shaped our behavior and our social identities, and how our experiences affect our values, beliefs, and worldview. Similarly, we need to critically examine our perspectives on differences, identifying any inaccuracies, stereotypes, or biases we may have about individuals or groups that differ from our own (Garcia & Ortiz, 2013).

Behaviorists align with objective knowing, valid, reliable, and firsthand accounts. Behavior analysts may need to shift how they think about objectivity because it is impossible to separate self from those under study. Fong et al. (2016) recommended that behavior analysts develop cultural awareness through first understanding their own cultural identity through a continual process of learning and growing. Also, the practice of reflexivity and incorporating positionality statements within qualitative research is another way behavior analysts can be cognizant of their contributions to the construction of meaning and lived experiences throughout the research process. We explore and address these considerations in depth in subsequent sections.

### Embrace Diversity

We must embrace the diversity within and between communities to gain a deeper understanding of the social world and how our research participants—particularly those who are multiply marginalized—navigate and interact within and across these communities. As Miller et al. (2019) states, “Our foundations allow hope for goals of social justice and cultural competence, responsiveness, and humility (p. 5).” However, it is unclear how ABA is embracing diversity and practicing cultural competence, responsiveness, and humility. To truly think intersectionally as ABA researchers, we must spend time learning about culture (our own and of our research participants) and seeking new experiences with potential participant groups of different cultures (Miller et al., 2019). We should critically reflect on our learning and experiences, especially those that challenge our assumptions, and allow them to thoughtfully inform and improve our practices over time. ABA has published specific frameworks and actionable steps borrowed and adapted from other fields to promote a self-reflective process to embracing diversity that may be helpful for readers to explore (see Beaulieu & Jimenez‐Gomez, 2022; Cirincione-Ulezi, 2020; Fong et al., 2016; Kirby et al., 2022; Mathur & Rodriguez, 2022; Wright, 2019 for examples).

### Learn With and From Participants

Research in ABA provides opportunities to learn with and from participants, and this does not mean learning only from the data as they relate to our research questions. To truly learn from participants, we include them and relevant invested parties throughout the entire research process. We intentionally and humbly seek input from diverse individuals (e.g., autistic, neurodiverse, culturally diverse, ethnically diverse, etc.) throughout the research process to center their voices. This approach aligns with community-based participatory research; it addresses the challenges of engaging invested parties in the research process, creating space to share knowledge and broaden discourse, shift power, and promote sustainment and use of programs (Wallerstein & Duran, 2010). Some implementation science frameworks also emphasize the importance of learning with and from participants and provide a guide to approach behavior change by addressing barriers that are important to society that can hinder intervention uptake (Cook & Odom, 2013; Fixsen et al., 2005; Odom et al., 2019). Implementation science is concerned with bridging the research-to-practice gap and emphasizes the importance of engagement with participants and invested parties to fully realize the benefits of research-validated interventions at scale.

### Seek a Collaborative Experience

Esposito and Evans-Winters’ (2021) emphasis on the importance of seeking collaborative relationships with participants is not a new recommendation to ABA. Collaboration extends learning with and from participants to bring together diverse perspectives to gain a deeper understanding of complex problems resulting in the creation of integrative solutions that go beyond individual viewpoints. We can draw from the literature on interdisciplinary collaboration involving applied behavior analysts to enhance our collaborative relationships with participants within intersectional research (e.g., Gasiewski et al., 2021; LaFrance et al., 2019; Slim & Reuter-Yuill, 2021). Just as each professional that an applied behavior analyst may work with has a wide range of knowledge, skills, and experience, so do the individuals participating in our studies. Overall, the literature in ABA emphasizes an important element of collaboration—increasing mutual understanding and respect. Fawcett (1991a, 1991b) outlines ten values to guide community research and action, and the first value is exactly this point. Fawcett offers several questions to assist with strengthening a collaborative relationship with participants:

(a) Are participants’ view of the community and its goals represented in the research goals, along with the researchers’ perspective?; (b) Is the community’s influence evident in the identification or choice of new research questions that are not suggested by either the discipline or the researchers’ past choices of topics?; (c) Does the method system require that the researcher become sufficiently knowledgeable about local ways by participating in activities of local origin (not just research activities) before, during, and after data collection?; (d) If an intervention is used, is it designed, adapted, and implemented in collaboration with participants?; (e) Is the work responsive to an initiative from the community, does it encourage such initiative, and do community members describe the research and action goals as their own? (p. 629)

In summary, the aforementioned claims proposed by Esposito and Evans-Winters (2021) align with prior work in ABA and can be helpful for researchers as they work to embrace intersectionality in their research. To further embrace an intersectional qualitative research approach, we ask researchers to consider how intersectionality applies to all stages of the research process. The following section incorporates examples for ABA researchers while highlighting the importance of mentorship in qualitative inquiry, the practice of reflexivity, and considerations for dissemination.

## Intersectionality Applies to All Stages of Research

Intersectionality has gained recognition not only as a framework for understanding complex social identities but also as a critical theoretical approach (Few-Demo, 2014; Few-Demo et al., 2022). Hence, intersectionality is both a theory and a methodological tool to explore how various forms of oppression and power intersect and impact an individual’s life (Crenshaw, 1991; Esposito & Evans-Winters, 2021). Conducting intersectional qualitative research is not a routine process. “Qualitative inquiry from an intersectional perspective unashamedly and ardently concedes that individuals can be multiply situated in the world and, thus, the researcher must be prepared to accept complexity as part of the research process” (Esposito & Evans-Winters, 2021, p. 4). Further, no one method is ideal for endeavoring in intersectional research (Rice et al., 2019). No matter the method or approach you choose, intersectionality is applicable to all stages of the research process, from identifying meaningful and relevant research questions to the analysis and dissemination of results. The following sections provide key considerations for each stage of research.

Prior to conducting intersectional qualitative research within ABA, we urge interested readers to seek a deeper understanding and level of competence. Applied behavior analysts are not typically trained in qualitative methods. This is primarily due to a paradigmatic stance that values quantitative and single case methods. Therefore, mentoring in qualitative research is required. Behavior-analytic researchers will most likely need to look for mentorship in qualitative inquiry, which will be much more in depth than learning how to conduct a focus group or employ thematic analysis, for instance. Mentorship should be targeted and thorough, as qualitative research is a philosophical practice. Guyotte and Wolgemuth (2022) provide guidance for navigating this specific mentor–mentee relationship. In addition, applied behavior analysts can attend stand-alone qualitative inquiry workshops, conferences, and join qualitative special interest groups. Applied behavior analysts can also improve their theoretical understanding of qualitative research through reading classic texts that provide philosophical positions, questions, and debates—both historical and contemporary—within and between different theoretical frameworks. When pursuing any opportunity for learning, researchers should aim to gain more than just the technical skills of qualitative research—such as data collection and analysis. While mastering these procedural skills is essential for becoming a proficient qualitative researcher, they alone are insufficient. We advocate for a more comprehensive approach to learning, designing, and conducting qualitative research, one that considers not only the practical aspects but also the theoretical foundations and representational dimensions of the field.

### Study Conceptualization

Intersectionality should be considered early on during study conceptualization because it allows researchers to explore the impact of sociohistorical forces of marginalization and view participant identities as multifaceted and interconnected at every stage of the research process (Abrams et al., 2020). This investigation of the multifaceted social identities of participants and how their experience with varying systems of privilege and oppression shaped their repertoire is an inductive (rather than deductive) endeavor, aligning with a behavior-analytic approach to scientific inquiry.

Once a study is conceptualized, a researcher selects the research questions. Qualitative research questions using an intersectional analytical framework are open ended, focused on who, what, when, where, why, and how, and are not focused on causality. The research questions should be designed to examine and ultimately explain social processes from the perspective of your participants. For example, an applied behavior analyst might be conducting a study to examine the effects of a training protocol to support caregivers in implementing a research-validated intervention. In addition to an experimental research question that seeks to examine causation and the effects of the training protocol, an intersectional qualitative research question could ask how the intersecting identities and social positions of the caregivers influence their perspectives of the training protocol and its outcomes. The research questions will lead to the appropriate methodology.

There are a variety of qualitative methodologies to choose from. Again, we strongly encourage mentorship on this topic. Esposito and Evans-Winters (2021) provide a description of numerous methodologies discussed from an intersectional perspective that can also guide you within your mentored qualitative inquiry. The chosen methodology frames the design and specific methods implemented. The sampling strategy also requires an intersectional lens to consider participants from marginalized groups and collaboration partners as researchers plan the study. Researchers must be reflective of who is invited to participate, why, what the benefits are, the outcomes of the study, and how they could impact participants and collaborators.

Be aware that it will take time and energy to implement the chosen methodology to truly understand your participant’s perspectives as you, at the same time, partake in self-exploration with intersectionality in mind. When feasible, including community members as part of the study team can help increase team diversity and foster active collaboration and co-leadership with the community (Abrams et al., 2020). In addition, it is crucial to address power imbalances, expectations, potential benefits, and concerns in research–community relationships before implementing the study protocol. This can often be achieved through collaboration with community partners or the use of community advisory boards that participate in decision-making related to research methodology and dissemination prior to the commencement of any research (Bailey et al., 2019).

### Data Collection

Data collection within qualitative inquiry can take many forms, including but not limited to interviews, focus groups, observations, and document analysis. Data collection that reflects intersectionality incorporates methods that center the experiences and worldviews of marginalized individuals (Esposito & Evans-Winters, 2021). Because researchers will have the most contact with participants at this stage, it is imperative that they practice as an intersectional researcher who is self-aware and can establish rapport with participants and build trust while centering the participants’ lived experience and knowledge. Esposito and Evans-Winters (2021) propose questions to reflect on the relationship with participants in the study that we would like to highlight: (1) What is the goal of the research, and who stands to benefit from it?; (2) What benefits might participants and collaborators receive (tangible and intangible) for participation and what benefits might you receive as the research?; (3) What are participants being asked to do and how will requests impact them emotionally, physically, or intellectually?; and (4) How have participants entrusted researchers with their stories and how can their stories be protected while portraying them justly? In the above example of a study examining the effects of a training protocol to support caregivers in implementing a research-validated intervention, data collection could expand beyond direct observation of caregiver implementation of the intervention (the experimental dependent variable) to also include interviews or focus groups with caregivers to explore how their intersecting identities might impact the degree to which the research-validated intervention, caregiver training procedures (the independent variable), and outcomes are meaningful and socially and ecologically valid. The researcher might also consider what other aspects of the caregiver’s identity place them in a vulnerable position relative to the researcher (i.e., power differentials), as the researcher’s relationship with the participants can impact data collection. If the researcher establishes a trusting relationship with participants, they are more likely to receive data that is authentic and will yield valid information about the intervention.

An established practice in qualitative research, called reflexivity, involves continual critical self-reflection while considering your multiple social identities and structures of power and ways in which the researcher, the participants, and the context influence each other (Abrams et al., 2020; Esposito & Evans-Winters, 2021). As Rice et al. (2019) argue, doing justice to intersectionality in research requires methodological rigor and reflexivity to authentically engage with the complexities of participants’ intersecting identities and lived experiences. A self-reflective intersectional qualitative researcher must examine power differentials, perceptions of race, and social identities from their own and participant’s perspectives, and how marginalized individuals experience systems of oppression. This practice occurs at the beginning and throughout the research process and allows researchers to hold awareness and reflect on power dynamics to play an equitable role in the participant–researcher relationship. Although a researcher may not be able to precisely identify how they have shaped the research study, including a positionality or reflexivity statement may enable readers to contextualize the research and decide how to interpret the findings (Parson, 2019). Positionality or reflexivity statements promote the methodological rigor of the study to help ensure that findings are considered trustworthy and credible (Leko et al., 2021). Positionality statements should not be performative; the statement should include how the authors’ identity can shape their approach, perspective, and the overall research process. Including aspects of the author’s identity that are most relevant to the study can provide valuable context and understanding of how personal experiences, cultural background, social position, and biases may influence the execution of the research (King, 2024; Parson, 2019).

Additionally, several other well-established practices in qualitative research methods can be especially valuable for researchers looking to incorporate intersectionality into their work. For instance, piloting interview guides with individuals from the target population can help ensure that participants understand the questions and that the questions generate relevant information for the overarching research objectives (Patton, 2015). Collaborating with community members about ideal data collection sites, timing, and compensation for participation can also be beneficial. In this context, community-based participatory research (CBPR) approaches are particularly useful for designing research that is attuned to the needs and experiences of participants, helping researchers avoid common challenges that can hinder study progress or compromise data quality. Moreover, CBPR encourages an intersectional approach, allowing experiences to be understood within the community context, rather than in comparison to dominant societal norms (Bailey et al., 2019).

### Intersectional Analysis of Qualitative Data

As with quantitative data collection, qualitative data analysis can take many forms. We stress that qualitative data analysis requires training and mentorship and we recognize that these processes can seem peculiar to behavior analysts as they may not follow prescribed steps like quantitative data analysis. Further, it is important to recognize that qualitative data analysis methods are not intended to support a functional relation, causal relationships, or large-scale generalizability. The general qualitative data analysis process includes organizing the data, reading your data and writing notes or memos, describing, classifying, and interpreting the data, and representing the data (e.g., text, figure, diagram; Cresswell, 2013). The process depends on your approach to inquiry (e.g., grounded theory, ethnography, phenomenological, case study).

Data analysis using any approach can be enhanced by applying an intersectional framework. There is more than one way to do intersectional analysis (Christensen & Jensen, 2012). Hence, the following considerations are not universal but are meant to inspire the application of intersectional analysis. Researchers can take an inductive (from data to theory) or deductive (from theory to data) approach to analysis, although generally an inductive approach is used. For instance, an inductive approach may be appropriate to develop categories when a limited amount of research exists focused on understanding the lived realities of intersectionality (Thomas et al., 2021), while a deductive approach within an intersectional framework may originate from known social and historical contexts connected to oppression in participants’ stories (Abrams et al., 2020). It is imperative to analyze data in a way that honors the intersecting construction of categories and relations to power structures (Anthias, 2013) and how categories can differ across time and place (Rice et al., 2019). Since constructions of categories vary widely across societies, researchers must consider the specific context in which the categories are constructed and know that the meaning of a category can also differ for individuals within it (Rice et al., 2019). Researchers can also consider analysis of context as certain identities or social constructs may be more or less salient in different contexts (Anthias, 2013). For example, within a study involving caregiver participants, analysis of interview data could include the impact of culture on the appropriateness and effectiveness of the intervention from the unique perspective of participants and their context. Overall, it is important to be clear about the levels of analysis used to uphold the promise of an intersectional approach to qualitative data analysis (e.g., individual level, interactional level considering interpersonal interactions, and at a structural level connecting lived experiences to broader systemic factors; Anthias, 2013).

Rigorous reflexive practices should be employed throughout the analysis process, as well as during the subsequent interpretation and reporting stages. It is important to acknowledge that bias may arise from the interaction between the data and the researchers’ backgrounds. Care must be taken to prevent the unintentional reinforcement or perpetuation of existing social inequalities and biases during the analysis process. The APA 7th Edition Publication Manual (2020) includes guidelines for authors regarding bias-free language with a section focused specifically on intersectionality. This guidance urges authors to be sensitive to the dimensions of identity and social systems as they write about the personal characteristics of their participants and in their reporting and interpretation of the results. Researchers are also urged to note the impact of any intersections on their findings as relevant to the study (APA, 2020).

### Dissemination

Intersectionality is also relevant in the dissemination of research results. Successful dissemination is achieved when a program, intervention, or practice has been adopted by the relevant stakeholders or participants (Dietrich, 2018). Because being “applied” in ABA is to be in pursuit of social justice (Pritchett et al., 2022), we must engage in activities that are likely to result in adoption and produce socially significant change to improve the lives of the individuals and communities that participated in our study. Effective dissemination activities go beyond publication in academic journals and presentations at professional conferences, as these outlets are unlikely to reach the audience that can most benefit from what was learned through your study. The individuals and communities targeted in ABA studies may not actively seek out applied behavior analysts, no matter how grand the intervention or convincing the data are. “We built a better mousetrap but the world did not beat a path to our door. It is somewhat ironic that what is arguably a science of influence (behavior analysis) has not been more effective at influencing the adoption rate of a science of influence” (Dietrich, 2018, p. 541).

We want to be explicitly clear that the process of dissemination (and adoption) should not be centered with the aim of convincing someone to do something or to achieve “buy in.” This post-hoc approach disregards the need for honest, reciprocal, engagement with invested parties and participants throughout the research process. Rogers (2003) proposes variables that influence adoption and the process of how individuals and groups come to adopt or reject practices. He argues that for a practice to be adopted, it should not only be (1) more advantageous relative to current practices, (2) simple to understand and use, (3) available to try for a limited time, and (4) efficacious with results readily visible, but also (5) compatible with values, beliefs, experiences, and needs of the people that will use it. An intersectional qualitative framework provides a systematic process to assist us in understanding an individual or community’s values, beliefs, prior experiences, and needs—increasing the likelihood of successful dissemination (adoption) to improve their lives.

Publication is the main outlet of dissemination of our research. Publishing our work in ABA journals is important, and we understand the drive to publish in flagship journals like the *Journal of Applied Behavior Analysis*. However, it is imperative to also consider how your publication is or is not serving end users. Journals with an audience that may find practical utility in published work should be considered as this supports getting interventions widely implemented. Journals like *Behavior Analysis in Practice* draw more attention from practitioner-oriented audiences that can greatly increase the impact (Critchfield et al., 2023). We should also strive to publish our work beyond ABA journals to reach a broader scientific audience and address the broader societal issues that are woven into intersectional research. Our work often requires interdisciplinary training and collaborations (Kirby et al., 2022; Vincent et al., 2025). Therefore, we should consider publishing our work across disciplines. Open-access publications can also support the dissemination of this important work as the aim of open access is to make research outputs more accessible for a wider audience to read and use.

Publishing with community partners can assist us in reaching the communities we study, help promote a shared responsibility for dissemination and support the beneficial impact of our work. Co-authorship does not come without its challenges, especially within a system traditionally led by only researchers. Yet, frameworks, considerations, and lessons learned are available to support dissemination efforts when publishing with community partners (see Armer et al., 2022; Fursova, 2023; Holden et al., 2022 for examples). A collaborative exchange with community partners following publication to promote the work can also be an effective dissemination strategy (Geller, 2022). That is, community partners can offer researchers advice for disseminating their work while researchers can offer community members evidence-based information and support to enhance their implementation (Geller, 2022).

Presentations are another common outlet for dissemination of ABA work. However, presentations at ABA conferences tend to reach only a specific audience within the scientific community. Researchers should consider face-to-face communication that targets a broader scientific community and the general public. Attend and present at conferences with interdisciplinary partners and consider volunteering and sharing findings locally by organizing or participating in local conferences whose target audience aligns with your work.

Intersectional work can also influence policy. Tools have been developed to apply intersectionality to public policy (Hankivsky et al., 2014). The most commonly used channels for disseminating social policy research to policymakers are print materials and personal communications (Ashcraft et al., 2020). Creating a policy brief shared online could also support dissemination efforts. There are many successful examples of online dissemination of behavior analysis to emulate (Kelly et al., 2019). An important consideration when disseminating broadly is communicating your research in plain language. Translating research findings while considering cultural and linguistic differences (Chen et al., 2010) and avoiding behavior analytic and technical terms is recommended as they can hinder dissemination efforts (Becirevic et al., 2016; Critchfield et al., 2017; Jarmolowicz et al., 2008; Kelly et al., 2019).

Lastly, consider storytelling as a way of communicating our research process and results (Dietrich, 2018; Hineline, 2018) and do so in an intersectional manner. See Hineline (2018) for examples of a variety of contexts and disciplines in which storytelling has been successful. By describing our work in an engaging way that reflects our understanding of and respect for the intersecting identities and experiences of participants and communities, we are more likely to communicate in a way that matters to the people that matter most. Information gleaned through intersectional qualitative research can provide us with rich content to incorporate into our storytelling.

We acknowledge that the contingencies in higher education do not support many of the aforementioned dissemination activities, and the “publish or perish” requirements for tenure and promotion persist. That being said, it is up to us as behavior analysts to create contingencies that will occasion meaningful dissemination behavior. To help change our world for the better, we must engage in activities that are more likely to result in the adoption of research-validated interventions. We recognize the challenges of dissemination and suggest applied behavior analysts implement strategies to avoid burnout, ensuring a resilient and enduring framework for ongoing dissemination (Kelly et al., 2019).

## Conclusion

In this conceptual manuscript, we present a case for integrating intersectional qualitative inquiry into ABA intervention research. By embracing this approach, behavior analysts can gain a more nuanced understanding of the complex interplay between systems of privilege and oppression that shape an individual’s learning history. This shift in perspective aligns with a commitment to social justice and equity, while also addressing critiques regarding aversive procedures and normalization. The use of qualitative methods, viewed through an intersectional lens, offers a pathway for ABA to remain relevant in addressing the diverse needs of the populations we serve. Furthermore, this approach can enhance our ability to design interventions that are more culturally responsive and socially just. As we move forward, it is crucial for behavior analysts to critically examine their practices and embrace methodologies that honor the lived experiences of individuals, particularly those from marginalized communities. By doing so, we can foster a more inclusive research approach that serves the best interests of all individuals.

## Data Availability

This manuscript has no associated data
